# Vitamin D Treatment Sequence Is Critical for Transcriptome Modulation of Immune Challenged Primary Human Cells

**DOI:** 10.3389/fimmu.2021.754056

**Published:** 2021-12-10

**Authors:** Henna-Riikka Malmberg, Andrea Hanel, Mari Taipale, Sami Heikkinen, Carsten Carlberg

**Affiliations:** ^1^ Institute of Biomedicine, University of Eastern Finland, Kuopio, Finland; ^2^ Institute of Clinical Medicine, University of Eastern Finland, Kuopio, Finland

**Keywords:** immune challenge, infection, lipopolysaccharide, β-glucan, PBMCs, vitamin D, transcriptome, responsive genes

## Abstract

Microbe-associated molecular patterns, such as lipopolysaccharide (LPS) and β-glucan (BG), are surrogates of immune challenges like bacterial and fungal infections, respectively. The biologically active form of vitamin D, 1α,25-dihydroxyvitamin D_3_ (1,25(OH)_2_D_3_), supports the immune system in its fight against infections. This study investigated significant and prominent changes of the transcriptome of human peripheral blood mononuclear cells that immediately after isolation are exposed to 1,25(OH)_2_D_3_-modulated immune challenges over a time frame of 24-48 h. In this *in vitro* study design, most LPS and BG responsive genes are downregulated and their counts are drastically reduced when cells are treated 24 h after, 24 h before or in parallel with 1,25(OH)_2_D_3_. Interestingly, only a 1,25(OH)_2_D_3_ pre-treatment of the LPS challenge results in a majority of upregulated genes. Based on transcriptome-wide data both immune challenges display characteristic differences in responsive genes and their associated pathways, to which the actions of 1,25(OH)_2_D_3_ often oppose. The joined BG/1,25(OH)_2_D_3_ response is less sensitive to treatment sequence than that of LPS/1,25(OH)_2_D_3_. In conclusion, the functional consequences of immune challenges are significantly modulated by 1,25(OH)_2_D_3_ but largely depend on treatment sequence. This may suggest that a sufficient vitamin D status before an infection is more important than vitamin D supplementation afterwards.

## Introduction

After infection or vaccination, cells of the innate immune system, such as monocytes in circulation and macrophages in tissues, show long-term changes in their epigenome, transcriptome and cell physiology (1). This reprogramming of immune cells can be induced by microbe-associated molecular patterns (2), i.e., by molecules that are preferentially or even exclusively found on the surface of microbes, such as the glycolipid LPS on the outer membrane of Gram-negative bacteria (3) or the polysaccharide BG in the cell wall of the fungus *Candida albicans* (4). Both LPS and BG induce in monocytes and macrophages signal transduction cascades that start at the pattern-recognition receptors TLR4 (Toll like receptor 4) (5) and CLEC7A (C-type lectin domain containing 7A) (6), respectively, use either kinases of the MAPK (mitogen-activated protein kinase) family or the RAF1 (Raf-1 proto-oncogene, serine/threonine kinase)/AKT1 (AKT serine/threonine kinase 1) pathways and end with well-known transcription factors, such as CREB1 (cAMP responsive element binding protein 1), AP1 (activating protein 1) and NF-κB (nuclear factor κB). Thus, LPS and BG serve as surrogates of bacterial and fungal infections and induce significant changes in the transcriptome of innate immune cells (7, 8). The functional consequences of this so-called trained immunity are an enhanced response to a re-stimulation with microbial molecules, an extended production of pro-inflammatory cytokines and the increased ability to eliminate infectious microbes (9, 10). Trained immunity is mostly beneficial to the host, but it may also become maladaptive in the context of sepsis or autoinflammatory disorders (11).

Vitamin D is a secosteroid that activates *via* its metabolite 1,25(OH)_2_D_3_ a transcription factor, the nuclear receptor VDR (vitamin D receptor) (12), i.e., in contrast to LPS and BG, 1,25(OH)_2_D_3_ has a direct effect on gene regulation (13). The main endocrine site of 1,25(OH)_2_D_3_ production are proximal tubule cells of the kidneys, but also a number of immune cells are able to produce the nuclear hormone for para- and autocrine purposes (14). The general role of vitamin D is to maintain energetic and survival homeostasis of VDR-expressing cells (15), while its main specific functions are calcium homeostasis for supporting bone mineralization (16) and a modulation of the immune system (17). *Via* the latter vitamin D efficiently reacts on infectious diseases (18) and at the same time it helps to avoid overreactions, such as in autoimmune diseases (19). The modulatory role of vitamin D on the function of the immune system as a whole, i.e., on innate and adaptive immunity, is beneficial to the host (20). In contrast, vitamin D deficiency often associates with increased rates of complications of infectious diseases, such as tuberculosis (21) or COVID-19 (22), chronic inflammation, such as in inflammatory bowel disease (23), and autoimmune diseases, such as the onset and progression of multiple sclerosis (24, 25).

Vitamin D and its metabolites as well as their synthetic analogs have not only a disease preventive potential (26) but are also used for the therapy of diseases, such as the autoimmune disorder psoriasis (27). In this study, we ask the question, whether on the level of the transcriptome of primary immune cells there is a difference between 1,25(OH)_2_D_3_ treatment before, during or after immune challenge by LPS or BG. An answer should enable to judge, whether it is critical to have a sufficient vitamin D status before, during or after experiencing an infection. We investigate the transcriptome of peripheral blood mononuclear cells (PBMCs) that were immediately after isolation stimulated with either LPS or BG in the presence or absence of 1,25(OH)_2_D_3_. PBMCs represent a natural mixture of monocytes, undifferentiated macrophages, natural killer (NK) cells, T and B cells, i.e., of cells of the innate and adaptive immune system, of which monocytes and macrophages are the most vitamin D-responsive cell types (28). The modulation of the immune challenge with 1,25(OH)_2_D_3_ was 24 h after, 24 h before or in parallel corresponding to an *in vivo* situation of vitamin D_3_ supplementation after, before or during an infection. The results indicate that the functional consequences of immune challenges are significantly modulated by 1,25(OH)_2_D_3_ but largely depend on treatment sequence.

## Materials and Methods

### PBMC Isolation

Blood samples were collected from a single healthy individual (male, age 56 years, body mass index 25.1, vitamin D status 87.6 nM 25-hydroxyvitamin D_3_ in serum), who gave written informed consent to participate in the study. All experiments were performed in accordance with relevant guidelines and regulations related to the VitDbol trial (NCT02063334, ClinicalTrials.gov). The research ethics committee of the Northern Savo Hospital district had approved the study protocol (#9/2014). PBMCs were isolated from freshly collected peripheral blood using Vacutainer CPT Cell Preparation Tubes with sodium citrate (Becton Dickinson) according to manufacturer’s instructions. Deconvolution of RNA-seq data from triplicate solvent-treated samples of each of the three models determined the relative amount of B cells (5.5%), T cells (49.1%), NK cells (19.4%), monocytes/macrophages (23.8%) and other cells (2.2%) within the pool of PBMCs.

### PBMC Culture

**Figure 1:**
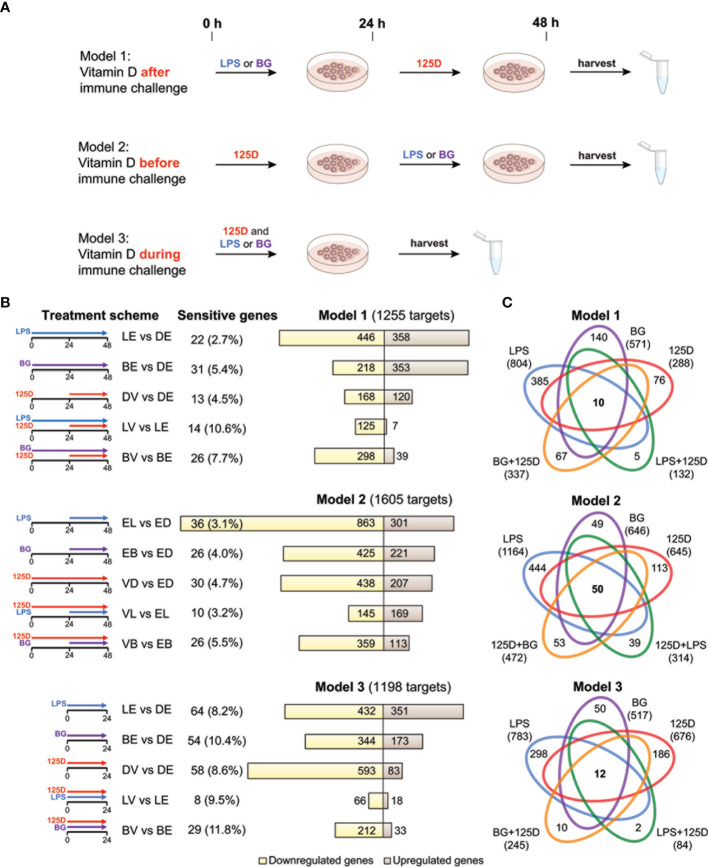
Transcriptomic changes of immune challenged PBMCs. PBMCs of one individual were isolated and treated in three repeats with 100 ng/ml LPS (L), 5 µg/ml BG **(B)** or solvent (0.1% DMSO (D)) in combination with 10 nM 1,25(OH)_2_D_3_ (V) or solvent (0.1% EtOH (E)) using three different models **(A)**. Freshly isolated PBMCs are stimulated with 1,25(OH)_2_D_3_ (125D) after (model 1), before (model 2) or during (model 3) immune challenge with LPS or BG. RNA is extracted and RNA-seq analysis indicates differentially expressed genes for the 15 different treatment conditions indicated by pictograms **(B)**. The number of cell culture sensitive genes is calculated in reference to the 165 differently regulated genes found between models 1 and 2 (for models 1 and 2) and the 152 differently regulated genes found between models 1 and 3 (for model 3) (**Figure S3B**). Bar charts monitor counts of up- (brown) and downregulated (yellow) genes for the indicated gene set comparisons. Venn diagrams display the overlap of different treatments within each model **(C)**. Gene numbers in brackets represent the total number of genes found responsive to the indicated treatment, while gene numbers in bold highlight common genes of all treatment conditions. Blue: LPS, purple: BG, red:1,25D, green: LPS/1,25D, orange: BG/1,25D.

PBMCs were washed with phosphate-buffered saline and immediately cultured at a concentration of 0.5 million cells/ml in 5 ml RPMI 1640 medium supplemented with 10% charcoal-depleted fetal calf serum, 2 mM L-glutamine, 0.1 mg/ml streptomycin and 100 U/ml penicillin. Cells were kept at 37 °C in a humidified 95% air/5% CO2 incubator. PBMCs were treated within one hour after taking them into culture with 100 ng/ml LPS (Sigma-Aldrich), 5 µg/ml β-1,3(D)-glucan (BG) (Sigma-Aldrich) or their solvent dimethyl sulfoxide (DMSO) (final concentration 0.1%) and 10 nM 1,25(OH)_2_D_3_ (Sigma-Aldrich) or its solvent ethanol (EtOH) (final concentration 0.1%) using three different models (**Figure 1A**). In model 1, cells were first exposed for 24 h to LPS, BG or DMSO and then either 1,25(OH)_2_D_3_ or EtOH were added for another 24 h without a wash-out step. In model 2, cells were first stimulated for 24 h with 1,25(OH)_2_D_3_ or EtOH and then for additional 24 h with LPS, BG or DMSO. In model 3, cells were incubated for 24 h simultaneously with LPS, BG or DMSO and 1,25(OH)_2_D_3_ or EtOH. Each *in vitro* experiment had been performed in three biological repeats within one week with cells from the same donor.

### RNA-seq Analysis

Total RNA was isolated using the High Pure RNA Isolation Kit (Roche) according to manufacturer’s instructions. RNA quality was assessed on an Agilent 2100 Bioanalyzer system (RNA integrity number ≥ 8). rRNA depletion and cDNA library preparation were performed using New England Biolabs kits NEBNext rRNA Depletion Kit, NEBNext Ultra II Directional RNA Library Prep Kit for Illumina and NEBNext Multiplex Oligos for Illumina (Index Primers Sets 1 and 2) according to manufacturer’s protocols. RNA-seq libraries went through quality control with an Agilent 2100 Bioanalyzer and were sequenced on a NextSeq 500 system (Illumina) at 75 bp read length using standard protocols at the Gene Core facility of the EMBL (Heidelberg, Germany).

The single-end, reverse-stranded cDNA sequence reads were aligned (without any trimming) to the reference genome (version GRCh38) and Ensembl annotation (version 93) using STAR (version 2.6.0c) with default parameters. Read quantification was performed within the STAR alignment step (–quantMode GeneCounts). Mapped and unmapped read counts are listed in **Table S1**. Ensembl gene identifiers were annotated with gene symbol, description, genomic location and biotype by accessing the Ensembl database (version 101) *via* the R package BiomaRt (version 2.44.1) (29). Gene identifiers missing external gene name annotation, genomic location or being mitochondrially encoded were removed from the datasets. When a gene name appeared more than once, the entry with the highest average number of counts was kept.

Differential gene expression analysis was computed in R (version 3.6.3) using the tool EdgeR (version 3.28.1) (30) that uses negative binomial distribution to model gene counts. The gene-wise statistical test for differential expression was computed using the generalized linear model quasi-likelihood pipeline (31). In order to mitigate the multiple testing problem, only expressed genes were tested for differential expression. The filtering threshold was adjusted to the expression of the low expressed but highly specific vitamin D responsive gene *CYP24A1* (cytochrome P450 family 24 subfamily A member 1). For this purpose, read counts were normalized for differences in sequencing depth to counts per million (CPM). Each gene needed to have an expression of > 0.5 CPM in at least 36 out of 54 samples, in order to be considered. This requirement was fulfilled by 16,861 genes. After filtering, library sizes were recomputed and trimmed mean of M-value normalization applied, in order to eliminate composition bias between libraries. The underlying data structure was explored by visualizing the samples *via* multidimensional scaling (MDS) (**Figure S1**). MDS was computed *via* EdgeR’s function plotMDS() in which distances approximate the typical log2 fold change (FC) between the samples. This distance was calculated as the root mean square deviation (Euclidean distance) of the largest 500 log2FCs between a given pair of samples, i.e., for each pair a different set of top genes was selected. The two principal factors distinguishing the samples’ expression profiles were the type of immune challenge and whether they were treated with 1,25(OH)_2_D_3_. Thus, the meaningful clustering of samples confirmed the similarity of the triplicates and demonstrates the effects of the treatments. In this line, a design matrix was constructed for the following pairwise comparisons: i) LPS/EtOH (LE) with DMSO/EtOH (DE) reference, ii) BG/EtOH (BE) with DE, iii) DMSO/1,25(OH)_2_D_3_ (DV) with DE, iv) LPS/1,25(OH)_2_D_3_ (LV) with LE and v) BG/1,25(OH)_2_D_3_ (BV) with BE. Trended negative binomial dispersion estimate was calculated using CoxReid profile-adjusted likelihood method and together with empirical Bayes-moderated quasi-likelihood gene-wise dispersion estimates used for generalized linear model fitting. The empirical Bayes shrinkage was robustified against outlier dispersions as recommended (31). Finally, quasi-likelihood F-test was applied to inspect, whether the observed gene counts fit the respective negative binomial model. Only genes with a false discovery rate (FDR) < 0.001 and an absolute FC > 2 were considered. Mean-Difference (MA) plots were generated with vizzy (version 1.0.0), (https://github.com/ATpoint/vizzy) to display the expression profile of each of the 15 comparisons (**Figure S2**).

### Data Analysis and Presentation

Relative cell type composition within the PBMC pool was estimated by deconvolution *via* the algorithm CIBERSORTx (32) using the default LM22 validated gene-signature matrix and gene expression data of solvent-treated samples of all three models. Estimations are based on 1000 permutations. Venn diagrams were created applying the webtool jvenn (33) (http://jvenn.toulouse.inra.fr) and Manhattan plots were produced in R by using packages ggbio (version 1.36.0) (34) and GenomicRanges (version 1.40.0) (35). Based on transcriptome-wide data pathway analysis was performed *via* the webtool Enrichr (36, 37) (https://maayanlab.cloud/Enrichr/) utilizing the Kyoto Encyclopedia of Genes and Genomes (KEGG) 2019 Human pathways (38). Adjusted P-values were employed for pathway ranking and the threshold < 0.001 was applied. Integrative database Genecards (https://www.genecards.org) was used for gene product locations and functions.

## Results

### Transcriptome Changes Due to Immune-Challenges or Vitamin D Stimulation

PBMCs of a single healthy individual were stimulated immediately after isolation with LPS, BG or solvent control (DMSO) in the presence of 1,25(OH)_2_D_3_ or its solvent (EtOH) (**Figure 1A**). Three different models were applied: in model 1 the cells were first exposed to LPS or BG for 24 h and then for another 24 h to 1,25(OH)_2_D_3_, in model 2 the sequence was changed, i.e., first 1,25(OH)_2_D_3_ stimulation for 24 h and then treatment with LPS or BG, and in model 3 immune challenges and 1,25(OH)_2_D_3_ were applied simultaneously for 24 h. The experiments of each model were performed in three repeats followed by RNA-seq and differential gene expression analysis. When the thresholds FDR < 0.001 and absolute FC > 2 were applied, 1255, 1605 and 1198 differentially expressed genes were detected in models 1, 2 and 3, respectively (**Table S2** and **Figure S3A**). For comparison, the influence of cell culture conditions like different treatment times (48 h in models 1 and 2 versus 24 h in model 3) were estimated by differential gene expression analysis of solvent-treated samples of each model (**Figure S3B**). These differences were largely model specific (75.1% of all) and only the five genes *ACP5* (acid phosphatase 5, tartrate resistant), *ALDH1A1* (aldehyde dehydrogenase 1 family member A1), *CCL24* (C-C motif chemokine ligand 24), *CD302* (cluster of differentiation 302) and *SPARC* (secreted protein acidic and cysteine rich) were identified as common genes that are sensitive to cell culture conditions.

In 13 of the 15 single and combined treatments the majority of the responsive genes were downregulated (**Figure 1B**). Within a given model, 23.6 to 33.4% of the responsive genes were downregulated in all treatments, while only 7.4 to 11.1% were exclusively upregulated. Thus, the majority (59.2 to 68.5%) of the responsive genes showed a mixed regulation profile (**Figure S3C**). In total of the three models, 1580 genes responded to LPS, 966 to BG and 1006 to 1,25(OH)_2_D_3_, from which 503, 388 and 201, respectively, have been previously reported (7, 39) (**Figure S3D**).

In all models, a treatment with LPS alone resulted in the highest count of responsive genes, while lowest numbers were obtained by a combined LPS/1,25(OH)_2_D_3_ treatment (**Figure 1C**). The number of responsive genes was also reduced by BG/1,25(OH)_2_D_3_ co-treatment but the effect was less prominent. LPS and BG showed 336, 505 and 375 overlapping genes in models 1, 2 and 3, respectively (**Figure S3E**). For comparison, in the presence of 1,25(OH)_2_D_3_ there were only 107, 177 and 57 common genes (**Figure S3F**). The count of 1,25(OH)_2_D_3_-responsive genes was only 288 in model 1, but 645 and 676 in models 2 and 3, respectively. Interestingly, the co-treatment with BG in model 1 increased the number of 1,25(OH)_2_D_3_-responsive genes, while in models 2 and 3 as well as in combination with LPS the numbers declined, i.e., the count and identity of vitamin D responsive genes was dependent on the co-treatment. The LPS treatment in model 2 is an exception, since in this case the ratio between up- and downregulated genes increased from 0.35 to 1.17 due to pre-treatment with 1,25(OH)_2_D_3_. The number of genes that are responsive to all three treatments, single and in combination, is rather low: 10 in model 1, 50 in model 2 and 12 in model 3 (**Figure 1C**). In contrast, there are 385, 444 and 298 genes that are in models 1, 2 and 3, respectively, exclusively responsive to the single treatment with LPS. These numbers are significantly higher than the counts for single treatments with BG (140, 49 and 50) or 1,25(OH)_2_D_3_ (76, 113 and 186).

In summary, the transcriptome of freshly isolated PBMCs shows in a time frame of 1-2 days significant (FDR < 0.001) and prominent (absolute FC > 2) changes in 1580 and 966 genes after immune challenges with LPS and BG, respectively, and in 1006 genes following 1,25(OH)_2_D_3_ treatment. The counts of the primarily downregulated LPS and BG responsive genes are clearly reduced to a total of 407 and 595, respectively, when the cells are treated 24 h after, 24 h before or in parallel with 1,25(OH)_2_D_3_. Interestingly, only a pre-treatment of the LPS challenge with 1,25(OH)_2_D_3_ leads to a majority of upregulated genes, while in the five remaining treatment protocols the proportion of downregulated genes even further increases.

### Key Genes and Pathways Representing Immune Challenge and Modulation by Vitamin D

**Figure 2:**
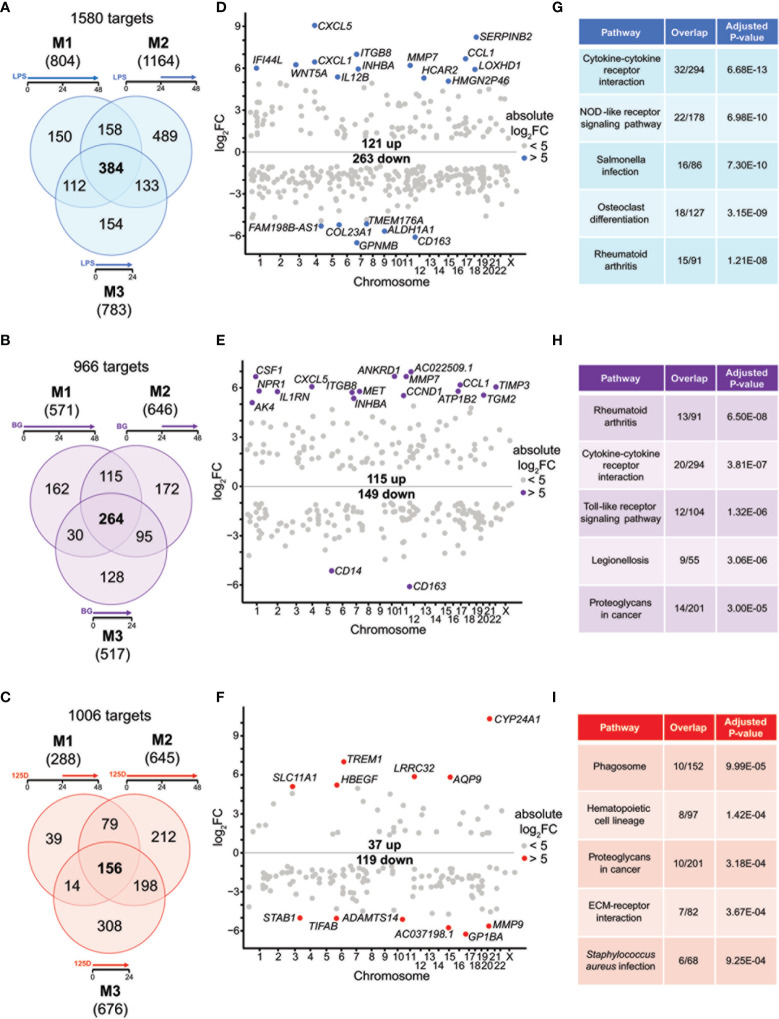
Genes and pathways affected by single stimulations. Venn diagrams display responsive genes obtained after single treatment with LPS **(A)**, BG **(B)** or 1,25(OH)_2_D_3_ (125D) **(C)** in all models. Gene numbers in brackets represent the total number of genes found responsive to the indicated treatment, while gene numbers in bold highlight common genes of all treatment conditions. Genome-wide distribution of overlapping genes is monitored by Manhattan plots of log2FC values from 48 h treatments, which are obtained from model 1 for LPS **(D)** and BG **(E)** and model 2 for 1,25(OH)_2_D_3_ **(F)**. Highly prominent (absolute log2FC > 5) responsive genes are named and marked by colored dots, whereas the other genes are indicated by grey dots. Top five KEGG pathways representing the most significantly enriched functions of the overlapping genes sorted by adjusted P-value **(G–I)**. Blue: LPS, purple: BG, red:1,25D. M1, model 1; M2, model 2; M3, model 3.

In order to identify key genes responding to either immune challenges by LPS or BG or 1,25(OH)_2_D_3_ modulation, we focused first on single treatments in all models. From the in total 1580 LPS responsive genes only 24.3% responded in all three models (**Figure 2A**). Similarly, only 27.3% of the 966 BG responsive genes (**Figure 2B**) and 15.5% of 1006 1,25(OH)_2_D_3_ responsive genes (**Figure 2C**) were common to all models. Thus, most responsive genes have a specificity for one or two models suggesting that the sequence of treatment has a major impact on the responsiveness of the cells.

For understanding the common aspects of the three models, we concentrated on joined responsive genes of the single treatments. Manhattan plots displayed the regular genome-wide distribution of the common responsive genes of LPS (**Figure 2D**), BG (**Figure 2E**) and 1,25(OH)_2_D_3_ (**Figure 2F**). The number of downregulated responsive genes was at all three treatment conditions higher than the count of upregulated genes. Despite the dominance of downregulation, the most prominent gene expression changes were observed for upregulated genes. Applying an absolute FC > 32 (= 25) threshold highlighted 19 LPS responsive genes (13 up and 6 down), 18 BG responsive genes (16 up and 2 down) and 12 1,25(OH)_2_D_3_ responsive genes (6 up and 6 down) (named in **Figures 2D–F**). The vast majority of these responsive genes are protein coding, but *HMGN2P46* is a pseudogene and *FAM198B-AS1*, *AC022509.1* and *AC037198.1* are non-coding RNA genes. Interestingly, the top responding genes indicated a number of common responsive genes for LPS and BG treatment [*CXCL5* (C-X-C motif chemokine ligand 5), *CCL1*, *CD163*, *ITGB8* (integrin subunit beta 8), *INHBA* (inhibin subunit beta A), *MMP7* (matrix metallopeptidase 7)] but no overlap with 1,25(OH)_2_D_3_ stimulation.

We used the transcriptome-wide data for pathway analysis using the webtool Enrichr with the 384, 264 and 156 common responsive genes of LPS, BG and 1,25(OH)_2_D_3_, respectively, pointed to their top five functions based on KEGG pathways. LPS treatment associated with “Cytokine-cytokine receptor interaction”, “Rheumatoid arthritis”, “NOD-like receptor signaling pathway”, “Salmonella infection” and “Osteoclast differentiation” (**Figure 2G**). The first two functions were also found with BG treatment, in addition to “Toll-like receptor signaling pathway”, “Legionellosis” and “Proteoglycans in cancer” (**Figure 2H**). The latter pathway was also associated with 1,25(OH)_2_D_3_ treatment alongside “Phagosome”, “Hematopoietic cell lineage”, “ECM-receptor interaction” and “Staphylococcus aureus infection” (**Figure 2I**). When the top five pathways were analyzed for each model separately (**Figure S4**), LPS treatment resulted for all models in “Rheumatoid arthritis” and “Osteoclast differentiation”, the functions “Cytokine-cytokine receptor interaction” and “NOD-like receptor signaling pathway” were found for models 1 and 3 and “Hematopoietic cell lineage” for models 1 and 2, while “Phagosome”, “Leishmaniasis” and “Influenza A” were model-specific (**Figures S2A–C**). BG treatment highlighted the pathways “Rheumatoid arthritis” and “Cytokine-cytokine receptor interaction” in all models, “Leishmaniasis” in models 2 and 3, while “Proteoglycans in cancer”, “Complement and coagulation cascades”, “ECM-receptor interaction”, “Hematopoietic cell lineage”, “Inflammatory bowel disease”, “Legionellosis” and “Salmonella infection” showed a model-specific fashion (**Figures S2D–F**). In contrast, 1,25(OH)_2_D_3_ triggered pathways in a more diverse way: “Phagosome”, “Staphylococcus aureus infection”, “Tuberculosis”, “Rheumatoid arthritis” and “Leishmaniasis” associated with two models, while “Hematopoietic cell lineage”, “Toxoplasmosis”, “Cytokine-cytokine receptor interaction”, “Osteoclast differentiation” and “Fluid shear stress and atherosclerosis” were found to be model-specific (**Figures S2G–I**).

Representative responsive genes were selected on the criteria i) being responsive to all treatments in at least one model ii) displaying prominent changes in expression and iii) being involved in the top KEGG pathways. The genes *TMEM176A* (transmembrane protein 176A), *WNT5A* (WNT family member 5A), *CXCL1*, *S100A8* (S100 calcium binding protein A8), *TNFSF15* (TNF superfamily member 15), *CSF1* (colony stimulating factor 1), *CD163*, *INHBA*, *CCL1*, *MMP9*, *CDKN1A* (cyclin dependent kinase inhibitor 1A) and *TREM1* (triggering receptor expressed on myeloid cells 1) all represent previously reported LPS, BG or 1,25(OH)_2_D_3_ responsive genes (7, 40, 41) (**Figure S5**). They represent a 4x3 matrix indicating that the whole group of responsive genes can be classified into 12 categories, such as being primarily responsive only to LPS or BG, both LPS and BG, or only 1,25(OH)_2_D_3_, as well being all down- or upregulated or showing a mixed response. This highlighted interesting specificities, such as that *CCL1* is clearly responsive both immune challenges but it barely responded to treatment with 1,25(OH)_2_D_3_, whereas *TREM1* showed distinct preference for 1,25(OH)_2_D_3_. The examples of the mixed regulation category indicated that immune challenges led to increased gene expression while 1,25(OH)_2_D_3_ showed opposite regulation. Furthermore, model-specific differences were observed, where, e.g., *TNFSF15* showed distinct responsiveness while *CSF1* responded almost the same in all models.

Taken together, the immune challenges LPS and BG display characteristic differences in responsive genes and the respective functions mediated by them, but also reasonable overlap in responding genes and regulated pathways. In contrast, 1,25(OH)_2_D_3_ primarily regulates a distinct set of genes and in case of joined responsive genes often show opposite direction of gene regulation. Despite these differences, all observed top functions relate to innate and adaptive immunity.

### Genes and Pathways Representing Vitamin D-Modulated Immune Challenges

**Figure 3:**
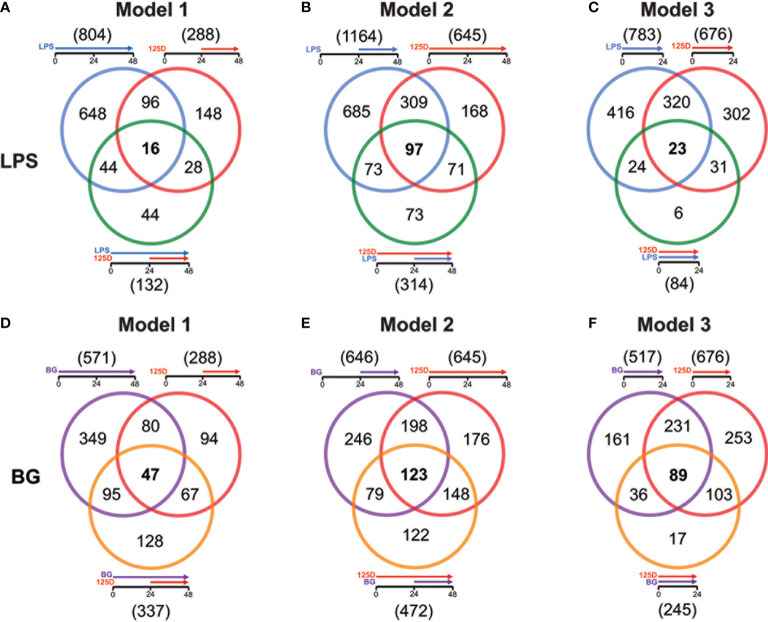
Genes responding to single treatment in relation to combined treatment. Venn diagrams display for the three models the overlap of genes responding to single treatment with LPS **(A–C)** or BG **(D–F)**, 1,25(OH)_2_D_3_ (125D) and the combination of both. Gene numbers in brackets represent the total number of genes found responsive to the indicated treatment, while gene numbers in bold highlight common genes of all treatment conditions. Blue: LPS, purple: BG, red: 1,25D, green: LPS/1,25D, orange: BG/1,25D.

For all models, the effects of either single treatments with LPS or BG and 1,25(OH)_2_D_3_ were compared with their respective combinations (**Figure 3**). In model 1, LPS and 1,25(OH)_2_D_3_ treatments overlapped in 112 genes, only 16 of which responded to the combined treatment of LPS and 1,25(OH)_2_D_3_ (**Figure 3A**). Individual LPS and 1,25(OH)_2_D_3_ treatments had in model 2 406 identical genes, 97 of which responded also to the combination of both treatments (**Figure 3B**). In model 3, LPS and 1,25(OH)_2_D_3_ treatments shared 343 genes, only 23 of which were found with their combination (**Figure 3C**). Similar results were obtained for immune challenge with BG, but compared to LPS the overlaps were larger: in model 1 127 BG and 1,25(OH)_2_D_3_ responsive genes overlapped, 47 of which in the context of dual stimulation (**Figure 3D**), in model 2 there were 321 identical genes, 123 of which responded to both stimuli (**Figure 3E**), and 320 shared genes in model 3, 89 of which occurred with both treatments (**Figure 3F**).

The combined treatments had reduced the total number of vitamin D responding genes to 407 in presence of LPS (**Figure S6A**) and 595 together with BG (**Figure S6B**). Interestingly, only 23 genes were commonly responding in all models to LPS/1,25(OH)_2_D_3_, while for BG/1,25(OH)_2_D_3_ the number was with 166 far higher. Furthermore, the model-specific combined responsive genes were in model 2 with 226 and 191 genes for LPS and BG co-treatment, respectively, clearly higher than in model 1 (66 and 94 genes) and model 3 (15 and 17 genes). Although model 2 had for combined LPS/1,25(OH)_2_D_3_ treatment a larger responsive gene count than models 1 and 3, only the pathways “ECM-receptor interaction” and “Cytokine-cytokine receptor” passed the threshold (**Figure S6C**). The latter function was also found in model 3, while all five top pathways of model 1 (“Phagosome”, “Proteoglycans in cancer”, “Legionellosis”, “Tuberculosis”, “Amoebiasis”) as well as the remaining four of model 3 (“Allograft rejection”, “Malaria”, “Rheumatoid arthritis” and “Pertussis”) were model-specific. In contrast, for the BG/1,25(OH)_2_D_3_ combination models 2 and 3 shared the top five pathways “Hematopoietic cell lineage”, “Phagosome”, “Tuberculosis”, “Cytokine-cytokine receptor interaction” and “Osteoclast differentiation” and model 1 at least the first three of them (**Figure S6D**). The two specific pathways of model 1 were “*Staphylococcus aureus* infection” and “Asthma”. Compared with the pathways highlighted by single treatments, the combined treatments relate more to infectious diseases and their specific pathogens.

Responsive genes serving as representative examples for the effects of combined treatments in comparison with single treatments (**Figure S7**) were selected by the same criteria as in case of the latter (**Figure S5**). The combined treatments showed either a boosting, inhibitory or mixed effect on gene expression. Moreover, genes were sorted by being under all conditions downregulated, upregulated or showing a mixed response providing each a 3x3 matrix for LPS and BG. Representative genes for LPS response were *FPR3* (formyl peptide receptor 3), *TGFBI* (transforming growth factor beta induced), *ITGB2* (integrin subunit beta 2), *CD14*, *FBP1* (fructose-bisphosphatase 1), *SEMA6B* (semaphoring 6B), *SLC22A23* (solute carrier family 22 member 23), *CXCL5* and *STAG3* (stromal antigen 3) (**Figure S7A**). The genes *TLR4*, *HLA-DRB5* (major histocompatibility complex, class II, DR beta 5), *CCL2*, *CLMN* (calmin), *IL1RN* (interleukin 1 receptor antagonist), *IL1R1* (interleukin 1 receptor type 1), *GAL3ST4* (galactose-3-O-sulfotransferase 4), *HBEGF* (heparin binding EGF like growth factor) and *G0S2* (G0/G1 switch 2) represent the BG response (**Figure S7B**). With exception of the genes HLA-*DRB5*, *SLC22A23*, *STAG3* and *GAL3ST4* the example genes are already known as LPS, BG and/or 1,25(OH)_2_D_3_ responsive genes (7, 39, 42).

In summary, the number of genes responding both to immune challenge and vitamin D, alone and in combination, indicate a descending ranking of models 2, 3 and 1. The joined response to BG and vitamin D shows a far better consensus between the models than that of LPS and vitamin D, both in gene count as well as by pathways. Responsive genes are either boosted or inhibited by dual treatments and often show mixed responses depending on the chosen model.

### Common and Specific Responses to Treatment Models

**Figure 4:**
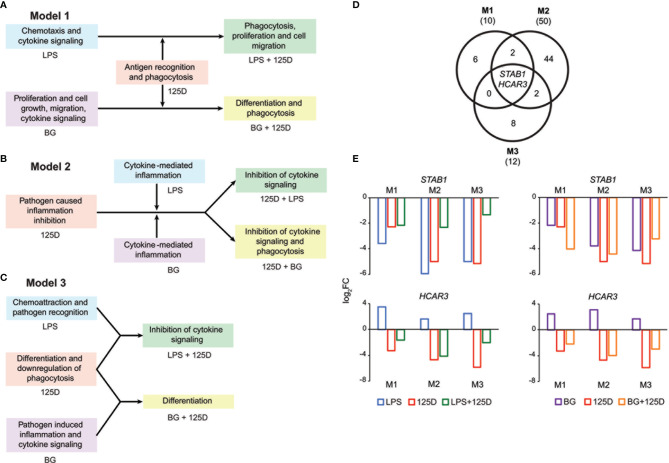
Consequences of single and combined treatments for common pathways and master genes. Key functions affected by single and combined treatments in model 1 **(A)**, model 2 **(B)** and model 3 **(C)**. A Venn diagram indicates the number of genes responding to the treatment combinations **(D)**. Gene numbers in brackets represent the total number of genes found responsive to the indicated treatment, while gene numbers in bold highlight common genes of all treatment conditions. Bar charts monitor the expression profiles of *STAB1* and *HCAR3* **(E)**. Blue: LPS, purple: BG, red: 1,25(OH)_2_D_3_ (125D), green: LPS/1,25D, orange: BG/1,25D. M1, model 1; M2, model 2; M3, model 3.

Integrating the functional consequences of the treatment sequence based on pathway analysis of single (**Figures 2G–I** and **S2**) and combined (**Figures S6C, D**) stimulation highlighted the differences of the three models. In model 1, immune challenge with LPS caused chemotaxis and induced cytokine signaling, whereas BG treatment affected proliferation, cell growth and cell migration but also increased cytokine signaling (**Figure 4A**). In contrast, stimulation with 1,25(OH)_2_D_3_-modulated genes and pathways involved in antigen recognition and phagocytosis. Interestingly, the combined treatment changed the effects of the immune challenges. The modulation of the LPS challenge with 1,25(OH)_2_D_3_ caused a shift towards phagocytosis, proliferation and cell migration, while the response to BG converted by modulation with 1,25(OH)_2_D_3_ into differentiation and phagocytosis. In model 2, the effects of all single treatments associated with inflammation, which in case of the immune challenges related to cytokines but with 1,25(OH)_2_D_3_ linked to pathogen inhibition (**Figure 4B**). Vitamin D modulated both immune challenges so that cytokine signaling was inhibited and in case of BG also phagocytosis was affected. In model 3, single treatment with LPS caused chemoattraction and affected pathogen recognition, while that of BG related to cytokine signaling and inflammation induced by pathogens (**Figure 4C**). In contrast, stimulation with 1,25(OH)_2_D_3_ alone affected differentiation and caused downregulation of phagocytosis, while in combination with LPS it inhibited cytokine signaling and together BG it initiated differentiation.

Only the genes *STAB1* (stabilin 1) and *HCAR3* (hydroxycarboxylic acid receptor 3) were in all models responsive to all types of treatments and serve as master examples for monitoring the differences between the models (**Figure 4D**). The *STAB1* gene encodes for a highly expressed membrane protein involved in endocytosis, which in every model was downregulated by all types of treatments (**Figure 4E**). The LPS/1,25(OH)_2_D_3_ co-treatment clearly reduced the change of downregulation being caused by respective single treatments. In contrast, the BG/1,25(OH)_2_D_3_ treatment resulted in model 1 in an enhanced change in downregulation, in model 2 in no significant effect and in model 3 in a slightly reduced change in downregulation. The *HCAR3* gene encodes for a G protein-coupled receptor with low affinity for nicotinic acid. In PBMCs the gene shows a low basal expression, was upregulated by both immune challenges but downregulated by 1,25(OH)_2_D_3_ and combined treatment. However, the combined treatments led to less change in downregulation than 1,25(OH)_2_D_3_ alone. Changes in *HCAR3* gene expression did not vary much between the three models, although in model 2 LPS had the lowest and BG the highest effect.

Taken together, a co-stimulation with 1,25(OH)_2_D_3_ is able to change the functional consequences of immune challenges but there are large differences as consequence of treatment sequence, i.e., of the chosen model. The genes *STAB1* and *HCAR3* are master examples monitoring the complex model-specific response to the modulation of immune challenges by vitamin D.

## Discussion

This study investigated on the level of significant (FDR < 0.001) and prominent (absolute FC > 2) changes of the transcriptome, whether 1,25(OH)_2_D_3_ stimulation affected transcriptional programing of primary human immune cells by immune challenges, such as the surrogates of bacterial and fungal infections, LPS or BG. Since there are ethical concerns against voluntary infections or *in vivo* treatments with LPS or BG, this study was designed *in vitro*, where PBMCs were treated immediately after isolation, in order to minimize transcriptional changes due to *in vitro* culture. Moreover, we retained from isolation of the most active and vitamin D responsive cell compartment (43), monocytes and undifferentiated macrophages, which together represent nearly a quarter of the PBMC population. Furthermore, we focused on the first 24-48 h after onset of stimulation, since transcriptional programming of the immune cells takes place within this time frame (7). Another important aspect in the design of this study was the sequence of treatment, where i) immune challenge before 1,25(OH)_2_D_3_ stimulation (model 1) mimicked the situation where an individual got infected at a low vitamin D status and vitamin D is used for treatment, ii) 1,25(OH)_2_D_3_ stimulation before LPS or BG treatment (model 2) represents infections at a high vitamin D status and iii) a simultaneous application of 1,25(OH)_2_D_3_ and LPS or BG (model 3) served as a reference. Nevertheless, this study has the limitation that not an isolated cell type was studied but a mixture of monocytes, macrophages, NK cells, and different subtypes of B and T cells. Moreover, the longer total treatment time of models 1 and 2 (48 h) limited the compatibility with model 3, in which the treatment was only for 24 h. However, models 1 and 2 were well comparable to each other. The focus of the study was on changes of the transcriptome, but its major findings need to be confirmed by proteome-wide approaches and functional assays, such as testing changes phagocytosis potential. Finally, humans have a personal vitamin D response index, i.e., they show inter-individual variations and respond with different strength to vitamin D_3_ supplementation (44). Therefore, the results of this study may not be generalized for the whole population.

The *in vitro* stimulated PBMCs showed to be most responsive to LPS (783 to 1164 responsive genes) and less affected by single treatments with BG (517 to 646 genes) and 1,25(OH)_2_D_3_ (288 to 676 genes). However, there are marked differences between the treatment models, so that in all three models only 384, 264 and 156 genes are responding to LPS, BG and 1,25(OH)_2_D_3_, respectively, while there are reasonable counts of model-specific responsive genes. For example, 489 LPS and 172 BG responsive genes are specific to model 2, while 308 1,25(OH)_2_D_3_ responsive genes are exclusively found in model 3. This is one important indication that the sequence of treatment has a large impact on the response of the transcriptome.

With the exception of BG treatment in model 1, the single treatments with LPS, BG and 1,25(OH)_2_D_3_ resulted in a majority of downregulated genes, i.e., all three stimuli rather diminish gene expression than enhance it. Moreover, the co-stimulations of the immune challenges with 1,25(OH)_2_D_3_ derived in a clearly reduced number of responsive genes, i.e., vitamin D appears to neutralize the responsiveness of a large number of LPS and BG responsive genes. Furthermore, with the exception of joint LPS/1,25(OH)_2_D_3_ stimulation in model 2, the co-treatments by vitamin D and the two immune challenges still mostly produced downregulated genes. It should be noted that the downregulation of a gene by one signal transduction pathway requires that first other signals upregulated of the gene. Thus, 1,25(OH)_2_D_3_-activated VDR seems to interfere with the responsiveness of many LPS and BG responsive genes, i.e., VDR counteracts to their mechanism of regulation. For example, 1,25(OH)_2_D_3_ and its receptor antagonize the pro-inflammatory actions of the transcription factors NF-κB (45). Interestingly, the interference of 1,25(OH)_2_D_3_ signaling with that of immune challenges does not require that the respective genes are primary vitamin D responsive genes, i.e., they do not have to contain VDR binding sites in their regulatory regions (46).

Although a rather large number of genes (112 to 406) respond in the different models to two different individual treatments, only 16 to 123 of these genes are responsive to the respective joint treatment. This is another indication that a co-treatment neutralizes the effects of the individual treatments. Nevertheless, model 2 displayed for both types of immune challenges a clearly higher number of genes with joint responsiveness than the two other models, i.e., the assumed beneficial effects of a pre-treatment with vitamin D are only in part neutralized by immune challenges. Interestingly, there are also cases where vitamin D and immune challenges boost each other. Since LPS, BG and 1,25(OH)_2_D_3_ mediate their signaling *via* different signal transduction pathways, it is not surprising that only two genes, *STAB1* and *HCAR3*, are in all models responsive to the three types of stimuli. The two genes serve as master genes demonstrating that the downregulation by 1,25(OH)_2_D_3_ affects their response to immune challenges. In case of the *STAB1* gene, 1,25(OH)_2_D_3_ reduces the amount of downregulation by LPS in all three models and it even further promotes the downregulation by BG in models 1 and 2. In contrast, the upregulation of *HCAR3* by LPS and BG is reversed by 1,25(OH)_2_D_3_ co-stimulation to a downregulation of the gene.

**Figure 5:**
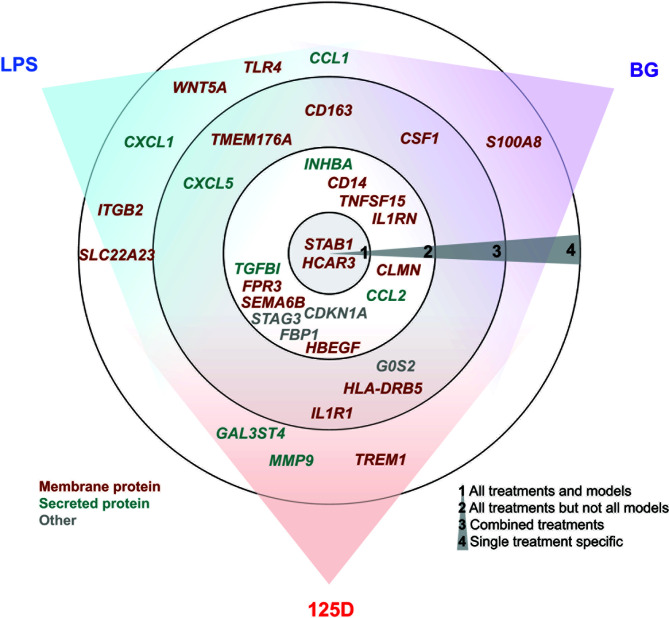
Summary of key responsive genes. LPS, BG and 1,25(OH)_2_D_3_ (125D) responsive genes are categorized based on their response to different treatments in different models. Circle 1: genes that are responsive to all treatments and models, circle 2: genes that are responsive to all treatments but not in every model, circle 3: genes that are responsive to at least two stimuli and their combination and circle 4: genes that are specifically responsive to stimuli. The gene positions in relation to colored sectors represent their preferences to the indicated stimuli. Genes are color-coded based on the main cellular location of their protein products. Blue: LPS, purple: BG, red: 1,25D.

In total, we selected 32 genes as representative examples for the different types of responses of PBMCs (**Figure 5**). The proteins encoded by these genes are located either within the plasma membrane (20/32) or are secreted (8/32). The majority of these proteins are either membrane receptors or cytokines and chemokines. Only two of the proteins, which are encoded by the representative genes, are found in the nucleus (*CDKN1A* and *STAG3*), whereas *FBP1* is located in the cytosol and *G0S2* in mitochondria. Most of the example genes are responsive to all treatments but not in all models. In contrast, some genes were only regulated by one stimulus, most of which are LPS responsive, while only *S100A8* is a specific responsive gene of BG. Interestingly, the example genes that are responsive to all treatments at least in one model show preference towards 1,25(OH)_2_D_3_ and BG or were equal between both. Out of the three applied treatments LPS signaling seems to be most independent. This is related to the fact that infection with bacteria carrying LPS on their surface are detrimental (47), while intake of vitamin D or BG are primarily beneficial (48, 49).

The stimulation of PBMCs with either LPS or BG affects the expression of genes that are involved in biochemical pathways of first line immune responses, such as enhancing cytokine signaling and inflammation. Furthermore, both immune challenges support pathogen recognition, but LPS has a focus on the extracellular and BG on the intracellular. In contrast, the stimulation of the cells with 1,25(OH)_2_D_3_ downregulates phagocytosis, induces differentiation and inhibits inflammation, i.e., pathways are activated that are rather contrary to those induced by immune challenges. While LPS and BG induce stress to cells and direct them to early responses like inflammation, vitamin D increases the potency of the immune system and boosts later steps in innate immune responses like destroying pathogens or initiating differentiation. Thus, the observed responses of PBMCs are most likely caused by their monocyte and macrophage compartment than by lymphocytes. When vitamin D is applied after immune challenge (model 1) both LPS- and BG-treated PBMCs initiate phagocytosis, but LPS-challenged cells activate proliferation and cell migration, while BG-treated cells differentiate. In contrast, a pre-treatment with vitamin D (model 2) reduces the activating effects of both LPS and BG on cytokine signaling as well as on inflammation and together with BG it activates phagocytosis. Interestingly, the simultaneous treatment with immune challenges and vitamin D (model 3) causes in case of LPS the inhibition of cytokine signaling and with BG the induction of differentiation. In all three models the co-treatment significantly changes the functional outcomes of immune challenges, which are directed towards disease- and pathogen-specific responses. Thus, the most disease-preventive reactions are caused by a pre-treatment with vitamin D (model 2).

In conclusion, this study provides a transcriptome-wide view how vitamin D modulates responses of the innate immune system to immune challenges like bacterial and fungal infections. A pre-treatment with vitamin D (model 2) appears to be more effective than its application after microbial challenges, such as infections with pathogens. Vitamin D_3_ supplementation will improve the vitamin D status of an individual and make vitamin D signaling *via* VDR and its target genes more effective. Since a large number of these responsive genes are involved in improving the functionality of immunity, their vitamin D-triggered activity can be considered as training of in particular the innate immune system (43). This suggests that vitamin D_3_ supplementation may have an important role in preventing infectious diseases or reducing their severe consequences.

## Data Availability Statement

The datasets presented in this study can be found in online repositories. The names of the repository/repositories and accession number(s) can be found in the article/**Supplementary Material**.

## Ethics Statement

The studies involving human participants were reviewed and approved by the research ethics committee of the Northern Savo Hospital District had approved the study protocol (#9/2014). The patients/participants provided their written informed consent to participate in this study.

## Author Contributions

H-RM and CC performed all experiments. AH performed differential gene expression analysis. H-RM performed data analysis. H-RM and CC wrote the manuscript, which was reviewed by AH, MT, and SH. All authors contributed to the article and approved the submitted version.

## Funding

CC had been supported by the Academy of Finland (grant No. 267067).

## Conflict of Interest

The authors declare that the research was conducted in the absence of any commercial or financial relationships that could be construed as a potential conflict of interest.

## Publisher’s Note

All claims expressed in this article are solely those of the authors and do not necessarily represent those of their affiliated organizations, or those of the publisher, the editors and the reviewers. Any product that may be evaluated in this article, or claim that may be made by its manufacturer, is not guaranteed or endorsed by the publisher.
