# Supplementary material for: Vitamin D Treatment Sequence Is Critical for Transcriptome Modulation of Immune Challenged Primary Human Cells
Source: Front Immunol. 2021 Dec 10;12:754056. doi: 10.3389/fimmu.2021.754056 (PMC8702862; doi:10.3389/fimmu.2021.754056)
Supplement: Supplementary file 1 [file DataSheet_1.pdf]

SUPPLEMENTARY DATA

SUPPLEMENTARY TABLES

Table S1: Read alignment. Total numbers and percentages of unmapped, multi-mapping, featureless, ambiguous and uniquely aligned reads per each sample are indicated. The raw sequencing reads were directly aligned, *i.e.*, no trimming was performed prior to alignment.

Table S2: Transcriptome changes of PBMCs. PBMCs of one individual were isolated and treated in three repeats with 100 ng/ml LPS (L), 5 μg/ml BG (B) or solvent (0.1% DMSO (D)) in combination with 10 nM 1,25(OH)$_2$D$_3$ (125D or V) or solvent (0.1% EtOH (E)) using three different models (**Fig. 1A**). RNA was extracted and subjected to RNA-seq analysis. In total of all three models the 2055 listed genes showed differential expression (thresholds FDR < 0.001 and absolute FC > 2) in at least one of the three models. For a given treatment condition data are shown only for those genes that passed the threshold. Tables of complete datasets of all expressed genes are found at GEO with accession number GSE172052. The discussed example genes (**Fig. 5**) are highlighted in red. Avg: average, SD: standard deviation, blue: L, purple: B, red: V, green: LV, yellow: BV, M1: model 1, M2: model 2, M3: model 3.

SUPPLEMENTARY FIGURES

Fig. S1: Sample quality assessment *via* MDS. Dimensionality reduction was applied using MDS, in order to visualize the similarities between the expression profiles of the 54 samples and to detect possible outliers and confounding effects. Distances on the plot approximate to the typical log_2FC between the samples. Thus, one unit represents a 2-fold change ($2^1=2$). Here, the two principal factors distinguishing the expression profiles were the type of immune challenge (dimension 1) and whether the samples were treated with $1,25(OH)_2D_3$ (dimension 2). Thus, MDS confirmed the similarity of the triplicates and demonstrates the effects of the treatments.

Fig. S2: Global effects of treatment. MA plots monitor the effects of treatment with LPS (L), BG (B) or solvent (DMSO (D)) in combination with $1,25(OH)_2D_3$ (V) or solvent (EtOH (E)) using three different models (**Fig. 1A**). The difference in expression change (log_2FC) for each tested gene is compared with the log_2 mean expression level between the compared groups (log_2CPM). Significantly (FDR < 0.001) up- and down-regulated genes are highlighted in red and blue, respectively. The horizonal red lines indicate the borders of absolute FC > 2, which was applied as additional threshold in the remaining study.

Fig. S3: Differential gene expression. Venn diagrams represent the overlap of responsive genes between the three models after treatment (**A**). Gene numbers in brackets represent the total number of genes found responsive to the indicated treatment, while gene numbers in bold highlight common genes of all treatment conditions. Taken together, 2055 different responsive genes were identified, of which 743 (36.2%) responded in all models, while 163 (model 1), 183 (model 3) and 449 (model 2) genes reacted exclusively in one model. The in total 795 model-specific responsive genes represent 38.7% of all responsive genes. When comparing the basal expression based on solvent controls (**B**), models 1 and 2 differed significantly (FDR < 0.001, absolute FC > 2) in 165 genes, models 1 and 3 in 152 genes and models 2 and 3 even in 435

genes. Please note that depending on the model and treatment 8 to 64 of the regulated genes (2.7 to 11.8%) were also found to be sensitive to cell culture conditions. In the three models responsive genes are categorized as down (red), mix (yellow) or upregulated (green) (**C**). Venn diagrams display the overlap of LPS, BG and 1,25(OH)$_2$D$_3$ (125D) responsive gene sets found in literature references [7, 38] and in this study (**D**) the overlap of LPS and BG responsive genes in the absence (**E**) and presence of 125D (**F**). Blue: LPS, purple: BG, red: 1,25D, green: LPS/1,25D, orange: BG/1,25D. M1, model 1; M2, model 2; M3, model 3.

Fig. S4: Top pathways triggered by single treatment. Top five KEGG pathways after single treatment with LPS (**A**, **B** and **C**), BG (**D**, **E** and **F**) or 1,25(OH)$_2$D$_3$ (125D, **G**, **H** and **I**) for all models are identified using Enrichr [36] pathway analysis and sorted by adjusted P-value. The bolded pathways are found from all three models with the respective treatment. Blue: LPS, purple: BG, red: 1,25D.

Fig. S5: Matrix of example genes representing single treatments. Bar charts monitor the expression profiles of 12 representative genes forming a 4x3 matrix concerning being preferentially responsive genes of LPS, BG, LPS and BG and 1,25(OH)$_2$D$_3$ (125D) as well as being down, mixed or upregulated. Blue: LPS, purple: BG, red: 1,25D. M1, model 1; M2, model 2; M3, model 3.

Fig. S6: Gene counts and top pathways triggered by combined treatment. Venn diagrams display for the three models the responsive gene sets of combined treatment by LPS and 1,25(OH)$_2$D$_3$ (125D) (**A**) or by BG and 125D (**B**). Gene numbers in brackets represent the total number of genes found responsive to the indicated treatment, while gene numbers in bold highlight common genes of all treatment conditions. Top five KEGG pathways (or those passing the threshold of adjusted P-value < 0.001) after combined treatment by LPS and 125D) (**C**) or by BG and 125D (**D**) for all models are identified using Enrichr [36] pathway analysis and

sorted by adjusted P-value. The bolded pathways are found from all three models with the respective treatment. Green: LPS/1,25D, orange: BG/1,25D. M1: model 1, M2: model 2, M3: model 3.

Fig. S7: Matrix of genes exemplifying combined treatments. Bar charts monitor for the three models the effects of all treatments on the sets of nine example genes of combined treatment of 1,25(OH)$_2$D$_3$ (125D) with LPS (**A**) or with BG (**B**), each set forming a 3x3 matrix concerning boosted, inhibited or mixed response as well as being downregulated, with mixed effects, or upregulated. Blue: LPS, purple: BG, red: 1,25D, green: LPS/1,25D, orange: BG/1,25D. M1: model 1, M2: model 2, M3: model 3.

Fig. S1

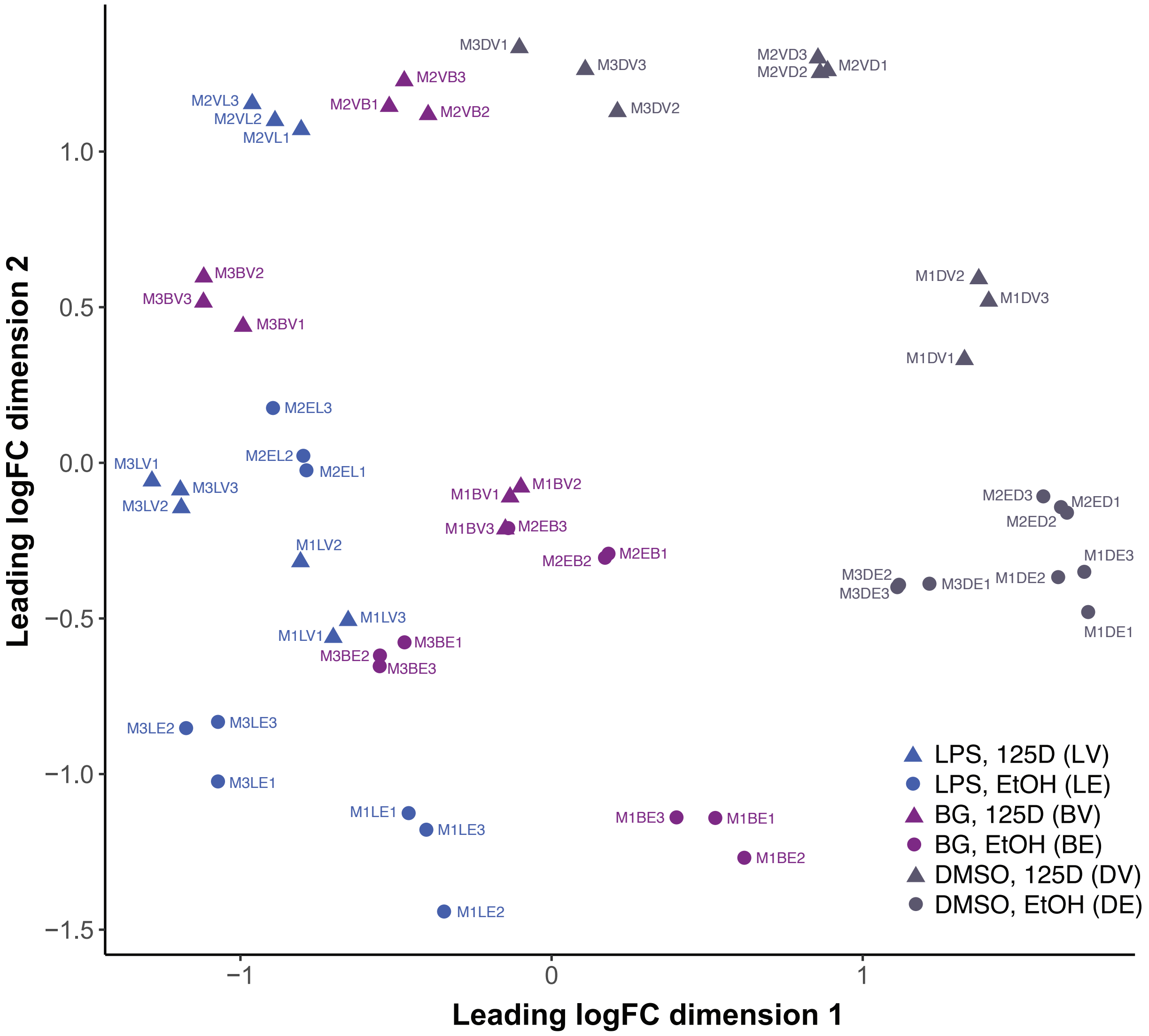

M3DV1
M3DV3
M2VD3
M2VD2
M2VD1
M2VB3
M2VL3
M2VB1
M2VB2
M3DV2
M2VL2
M2VL1
M3BV2
M3BV3
M3BV1
M1DV2
M1DV3
M1DV1
M2EL3
M2EL2
M2EL1
M3LV1
M3LV3
M3LV2
M1BV1
M1BV2
M1BV3
M2EB3
M2ED3
M2ED1
M2ED2
M2EB2
M2EB1
M1LV2
M3DE2
M3DE3
M3DE1
M1DE2
M1DE3
M1DE1
M1LV3
M1LV1
M3BE1
M3BE2
M3BE3
M3LE2
M3LE3
M3LE1
M1LE1
M1LE3
M1BE3
M1BE1
M1BE2
M1LE2
1.0
0.5
0.0
−0.5
−1.0
−1.5
−1
0
1
Leading logFC dimension 1
Leading logFC dimension 2
LPS, 125D (LV)
LPS, EtOH (LE)
BG, 125D (BV)
BG, EtOH (BE)
DMSO, 125D (DV)
DMSO, EtOH (DE)

Fig. S2

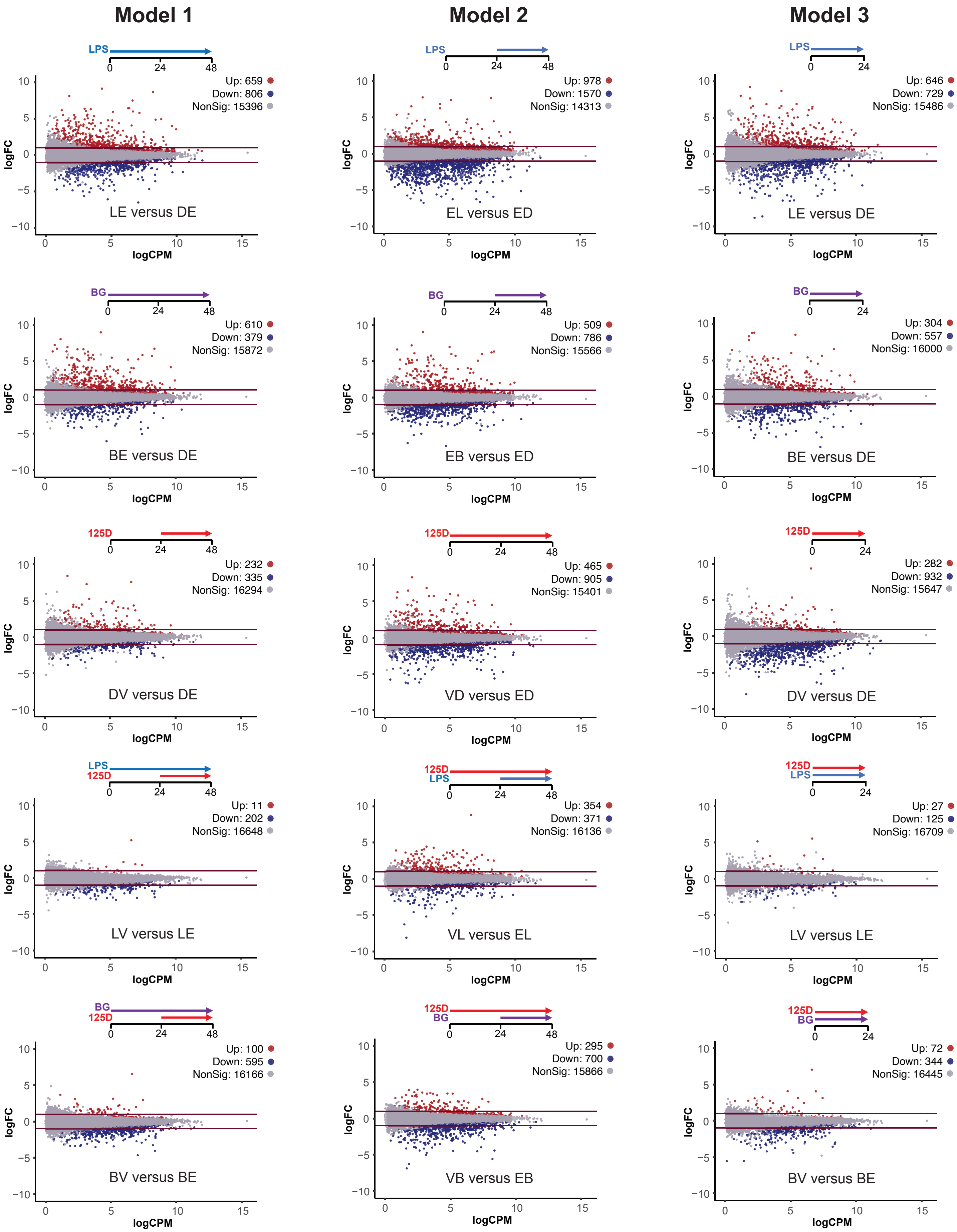

Model 1
Model 2
Model 3
LPS
0 24 48
Up: 659
Down: 806
NonSig: 15396
LE versus DE
LPS
0 24 48
Up: 978
Down: 1570
NonSig: 14313
EL versus ED
LPS
0 24
Up: 646
Down: 729
NonSig: 15486
LE versus DE
BG
0 24 48
Up: 610
Down: 379
NonSig: 15872
BE versus DE
BG
0 24 48
Up: 509
Down: 786
NonSig: 15566
EB versus ED
BG
0 24
Up: 304
Down: 557
NonSig: 16000
BE versus DE
125D
0 24 48
Up: 232
Down: 335
NonSig: 16294
DV versus DE
125D
0 24 48
Up: 465
Down: 905
NonSig: 15401
VD versus ED
125D
0 24
Up: 282
Down: 932
NonSig: 15647
DV versus DE
LPS
125D
0 24 48
Up: 11
Down: 202
NonSig: 16648
LV versus LE
125D
LPS
0 24 48
Up: 354
Down: 371
NonSig: 16136
VL versus EL
125D
LPS
0 24
Up: 27
Down: 125
NonSig: 16709
LV versus LE
BG
125D
0 24 48
Up: 100
Down: 595
NonSig: 16166
BV versus BE
125D
BG
0 24 48
Up: 295
Down: 700
NonSig: 15866
VB versus EB
125D
BG
0 24
Up: 72
Down: 344
NonSig: 16445
BV versus BE
logFC
logCPM

Fig. S3

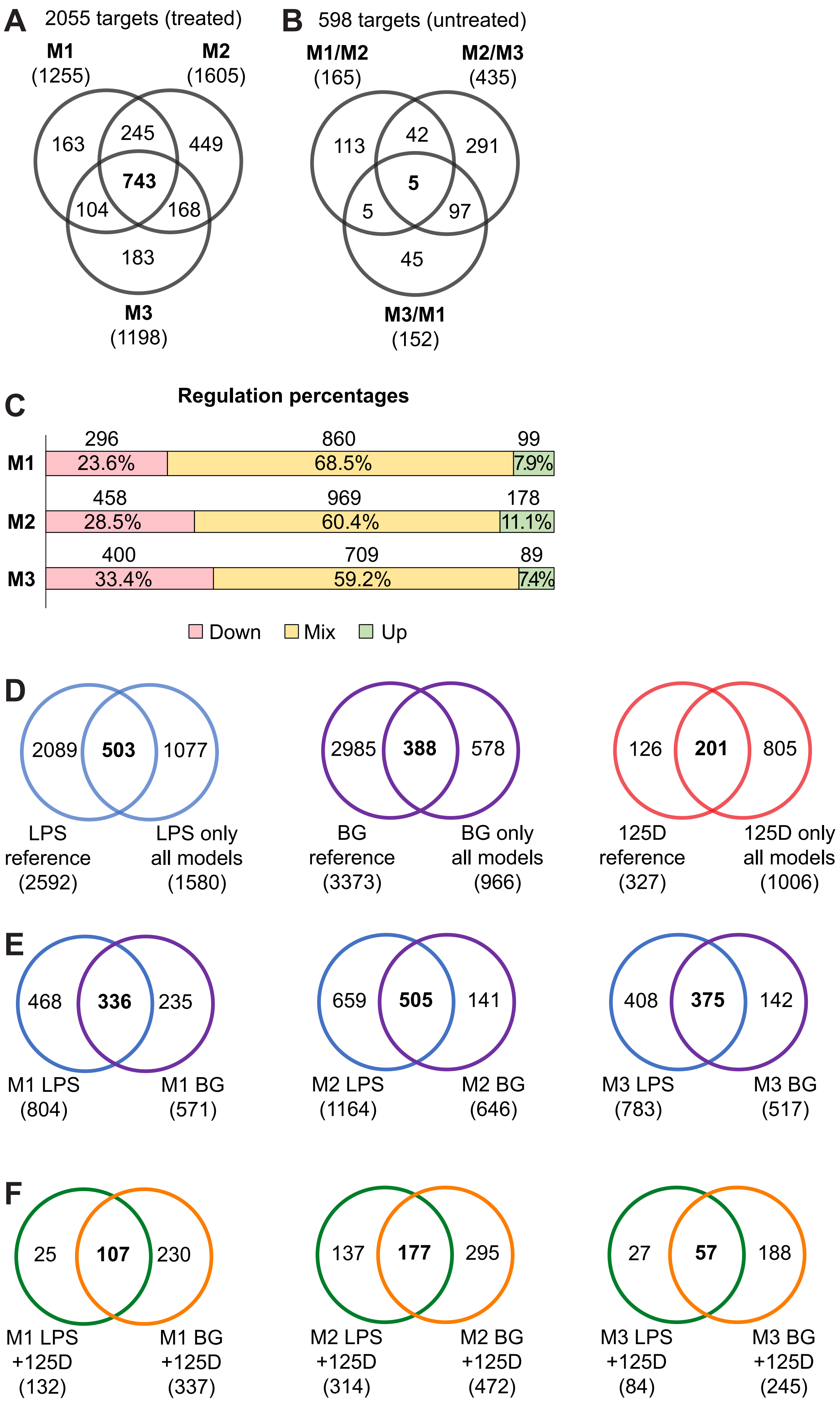

A
2055 targets (treated)
M1
(1255)
M2
(1605)
163
245
449
743
104
168
183
M3
(1198)
B
598 targets (untreated)
M1/M2
(165)
M2/M3
(435)
113
42
291
5
5
97
45
M3/M1
(152)
C
Regulation percentages
M1
296
23.6%
860
68.5%
99
7.9%
M2
458
28.5%
969
60.4%
178
11.1%
M3
400
33.4%
709
59.2%
89
7.4%
Down
Mix
Up
D
2089
503
1077
LPS reference (2592)
LPS only all models (1580)
2985
388
578
BG reference (3373)
BG only all models (966)
126
201
805
125D reference (327)
125D only all models (1006)
E
468
336
235
M1 LPS (804)
M1 BG (571)
659
505
141
M2 LPS (1164)
M2 BG (646)
408
375
142
M3 LPS (783)
M3 BG (517)
F
25
107
230
M1 LPS +125D (132)
M1 BG +125D (337)
137
177
295
M2 LPS +125D (314)
M2 BG +125D (472)
27
57
188
M3 LPS +125D (84)
M3 BG +125D (245)

Fig. S4

Model 1

A LPS

Pathway	Overlap	Adjusted P-value
Cytokine-cytokine receptor interaction	44/294	1.21E-11
Hematopoietic cell lineage	24/97	6.39E-11
**Rheumatoid arthritis**	23/91	8.03E-11
NOD-like receptor signaling pathway	31/178	3.20E-10
**Osteoclast differentiation**	25/127	1.92E-09

D BG

Pathway	Overlap	Adjusted P-value
Proteoglycans in cancer	25/201	1.71E-07
**Cytokine-cytokine receptor interaction**	28/294	3.46E-06
Complement and coagulation cascades	14/79	3.78E-06
ECM-receptor interaction	14/82	4.63E-06
**Rheumatoid arthritis**	14/91	1.26E-05

G 125D

Pathway	Overlap	Adjusted P-value
Hematopoietic cell lineage	18/97	5.67E-13
Phagosome	20/152	7.49E-12
Toxoplasmosis	17/113	4.15E-11
Staphylococcus aureus infection	14/68	4.15E-11
Tuberculosis	20/179	6.83E-11

Model 2

B LPS

Pathway	Overlap	Adjusted P-value
**Rheumatoid arthritis**	34/91	8.10E-17
Hematopoietic cell lineage	34/97	4.19E-16
**Osteoclast differentiation**	36/127	6.70E-14
Phagosome	38/152	7.18E-13
Leishmaniasis	26/74	1.46E-12

E BG

Pathway	Overlap	Adjusted P-value
**Rheumatoid arthritis**	29/91	5.76E-19
**Cytokine-cytokine receptor interaction**	39/294	8.39E-12
Leishmaniasis	20/74	1.08E-11
Hematopoietic cell lineage	22/97	2.46E-11
Inflammatory bowel disease	17/65	6.97E-10

H 125D

Pathway	Overlap	Adjusted P-value
Staphylococcus aureus infection	20/68	5.38E-12
Rheumatoid arthritis	21/91	1.16E-10
Leishmaniasis	19/74	1.21E-10
Tuberculosis	28/179	2.68E-10
Cytokine -cytokine receptor interaction	36/294	3.34E-10

Model 3

C LPS

Pathway	Overlap	Adjusted P-value
Cytokine-cytokine receptor interaction	54/294	3.12E-19
NOD-like receptor signaling pathway	37/178	6.24E-15
Influenza A	33/171	2.02E-12
**Osteoclast differentiation**	27/127	2.80E-11
**Rheumatoid arthritis**	23/91	2.80E-11

F BG

Pathway	Overlap	Adjusted P-value
Legionellosis	15/55	6.01E-10
Salmonella infection	18/86	6.01E-10
**Cytokine-cytokine receptor interaction**	32/294	6.01E-10
**Rheumatoid arthritis**	18/91	9.93E-10
Leishmaniasis	15/74	2.74E-08

I 125D

Pathway	Overlap	Adjusted P-value
Osteoclast differentiation	25/127	2.18E-10
Phagosome	25/152	6.82E-09
Leishmaniasis	17/74	2.61E-08
Rheumatoid arthritis	18/91	7.98E-08
Fluid shear stress and atherosclerosis	20/139	2.40E-06

Fig. S5

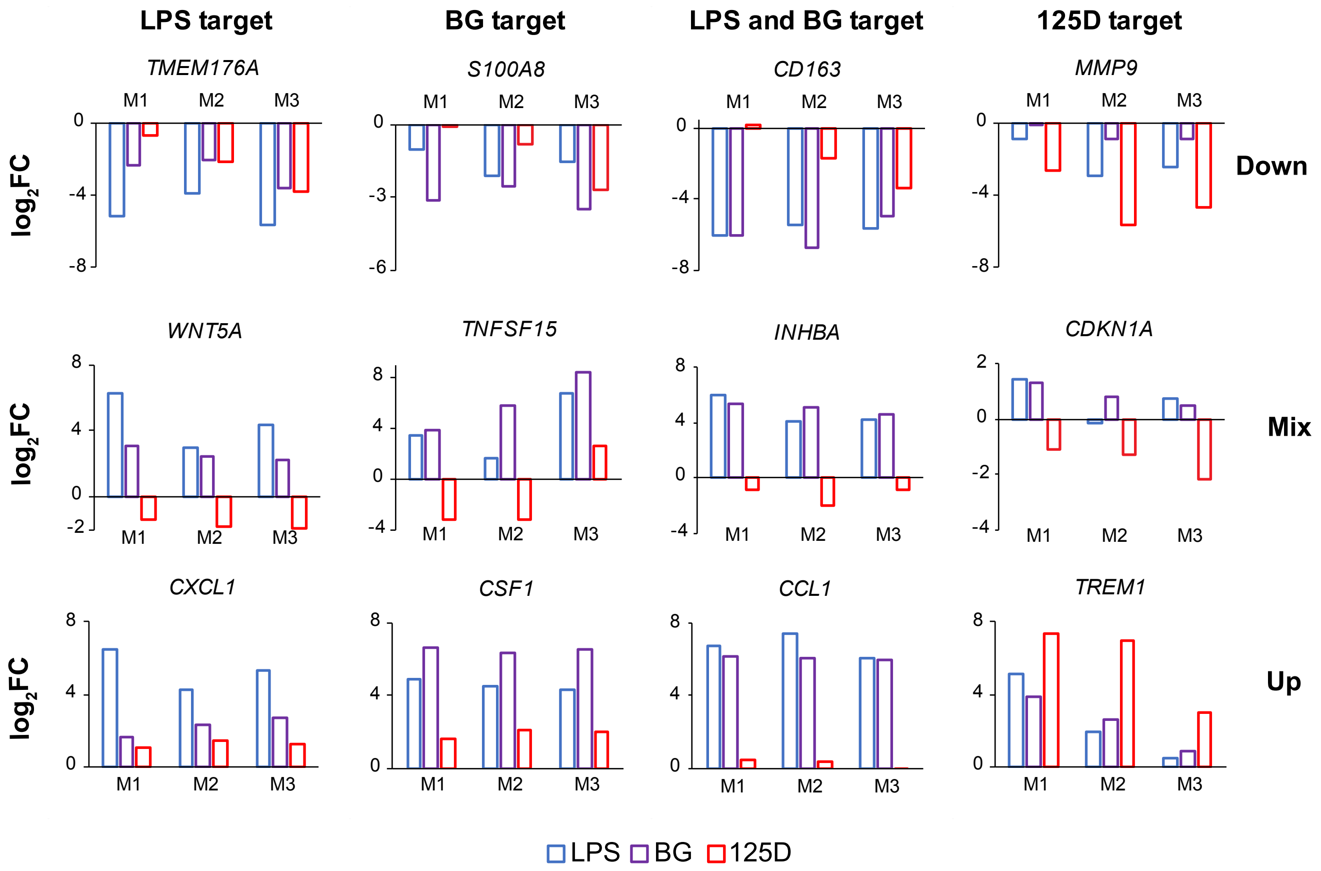
LPS target
BG target
LPS and BG target
125D target
TMEM176A
S100A8
CD163
MMP9
WNT5A
TNFSF15
INHBA
CDKN1A
CXCL1
CSF1
CCL1
TREM1
log2FC
M1
M2
M3
Down
Mix
Up
LPS
BG
125D

Fig. S6

A

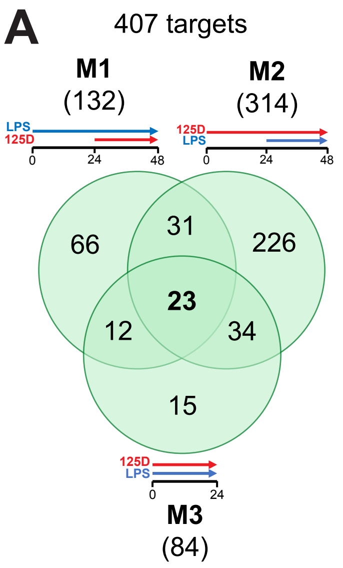

C

Model 1

Pathway	Overlap	Adjusted P-value
Phagosome	10/152	1.17E-05
Proteoglycans in cancer	10/201	7.83E-05
Legionellosis	6/55	9.45E-05
Tuberculosis	9/179	1.01E-04
Amoebiasis	7/96	1.01E-04

Model 2

Pathway	Overlap	Adjusted P-value
Cytokine-cytokine receptor interaction	23/294	5.80E-08
ECM-receptor interaction	9/82	5.46E-04
–	–	–
–	–	–
–	–	–

Model 3

Pathway	Overlap	Adjusted P-value
Cytokine-cytokine receptor interaction	16/294	1.15E-11
Allograft rejection	5/38	3.65E-05
Malaria	5/49	8.03E-05
Rheumatoid arthritis	6/91	8.03E-05
Pertussis	5/76	4.74E-04

B

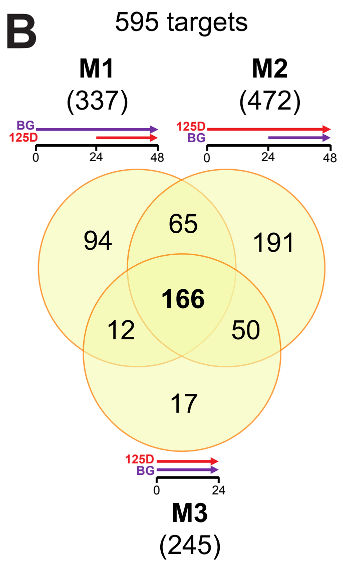

D

Model 1

Pathway	Overlap	Adjusted P-value
**Hematopoietic cell lineage**	18/97	8.39E-12
**Phagosome**	17/152	7.84E-08
Staphylococcus aureus infection	12/68	7.84E-08
Asthma	9/31	7.84E-08
**Tuberculosis**	17/179	3.81E-07

Model 2

Pathway	Overlap	Adjusted P-value
Cytokine-cytokine receptor interaction	32/294	1.32E-10
**Hematopoietic cell lineage**	19/97	1.32E-10
**Phagosome**	21/152	6.17E-09
Osteoclast differentiation	19/127	9.21E-09
**Tuberculosis**	22/179	1.32E-08

Model 3

Pathway	Overlap	Adjusted P-value
Osteoclast differentiation	14/127	1.16E-07
**Hematopoietic cell lineage**	12/97	2.68E-07
**Tuberculosis**	13/179	2.41E-05
**Phagosome**	11/152	1.53E-04
Cytokine-cytokine receptor interaction	15/294	1.61E-04

Fig. S7

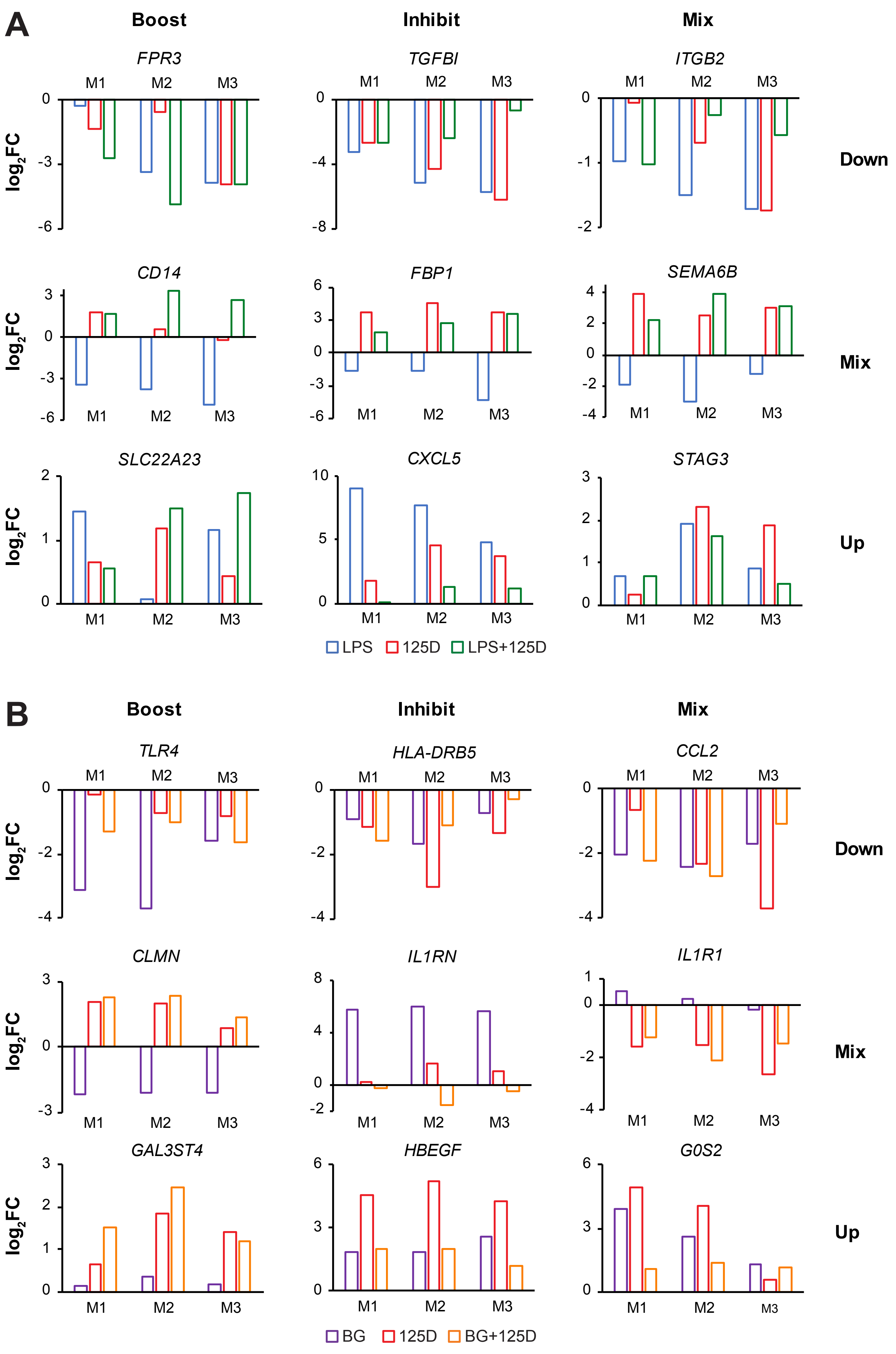
A
Boost
Inhibit
Mix
FPR3
TGFBI
ITGB2
CD14
FBP1
SEMA6B
SLC22A23
CXCL5
STAG3
M1
M2
M3
log_2FC
Down
Mix
Up
LPS
125D
LPS+125D
B
Boost
Inhibit
Mix
TLR4
HLA-DRB5
CCL2
CLMN
IL1RN
IL1R1
GAL3ST4
HBEGF
G0S2
BG
125D
BG+125D
